# Phytochemistry and Biological Activities of *Guarea* Genus (Meliaceae)

**DOI:** 10.3390/molecules27248758

**Published:** 2022-12-10

**Authors:** Wahyu Safriansyah, Siska Elisahbet Sinaga, Unang Supratman, Desi Harneti

**Affiliations:** 1 Department of Chemistry, Faculty of Mathematics and Natural Science, Universitas Padjadjaran, Sumedang 45363, Indonesia; 2 Central Laboratory, Universitas Padjadjaran, Sumedang 45363, Indonesia

**Keywords:** *Guarea*, Meliaceae, sesquiterpenoids, biological activities, phytochemistry

## Abstract

*Guarea* is one of the largest genera of the American Meliaceae family, consisting of over 69 species which are widely distributed in Mexico, Argentina, and Africa and are used in traditional medicine for several diseases. Previous studies reported that the *Guarea* species produce secondary metabolites such as sesquiterpenoid, diterpenoid, triterpenoid, limonoid, steroid, and aromatic compounds. The preliminary chemical investigation commenced by isolating the limonoid compound, dihydrogedunin, in 1962; then, 240 compounds were obtained from the isolation and hydrodistillation process. Meanwhile, sesquiterpenoid is a significant compound with 52% of *Guarea* species. The extract and compounds were evaluated for their anti-inflammation, antimalarial, antiparasitic, antiprotozoal, antiviral, antimicrobial, insecticidal, antioxidant, phosphorylation inhibitor, and cytotoxic biological activities. The *Guarea* genus has also been reported as one of the sources of active compounds for medicinal chemistry. This review summarizes some descriptions regarding the types of *Guarea* species, especially ethnobotany and ethnopharmacology, such as the compounds isolated from the part of this genus, various isolation methods, and their bioactivities. The information can be used in further investigations to obtain more bioactive compounds and their reaction mechanisms.

## 1. Introduction

The Meliaceae or mahogany family is distributed in tropical and subtropical regions such as Himalaya, South and Central America, Africa, as well as South and Southeast Asia. They consist of over 579 species and 51 genera with the major secondary metabolites being terpenoids and limonoids along with minor compounds such as flavonoids, lignans, chromones, and phenolics [1]. The biological activities of the Meliaceae family include cytotoxic [2,3,4,5,6], antiviral [7,8,9,10], antiplasmodial [11,12,13,14], antioxidant [15,16,17,18], antimicrobial [19,20,21,22], antifeedant [23,24,25,26], and anti-inflammation [27,28,29,30,31].

*Guarea* is one of the largest genera of the American Meliaceae family consisting of over 69 species widely distributed in Mexico and Argentina [32], while a few species are found in Africa [33]. Initial chemical investigation which commenced in 1962 by Housley et al. [34] isolated a limonoid compound, dihydrogedunin (221), from the ground heartwood of *G. thompsonii* (Nigerian pearwood). Subsequently, eight classes of secondary metabolites have been identified along with their biological activities, such as cytotoxic, anti-inflammation, antimalarial, antiparasitic, antiprotozoal, antiviral, antimicrobial, insecticidal, antioxidant, and phosphorylation inhibitor.

## 2. Methodology and Botany

**Figure 1:**
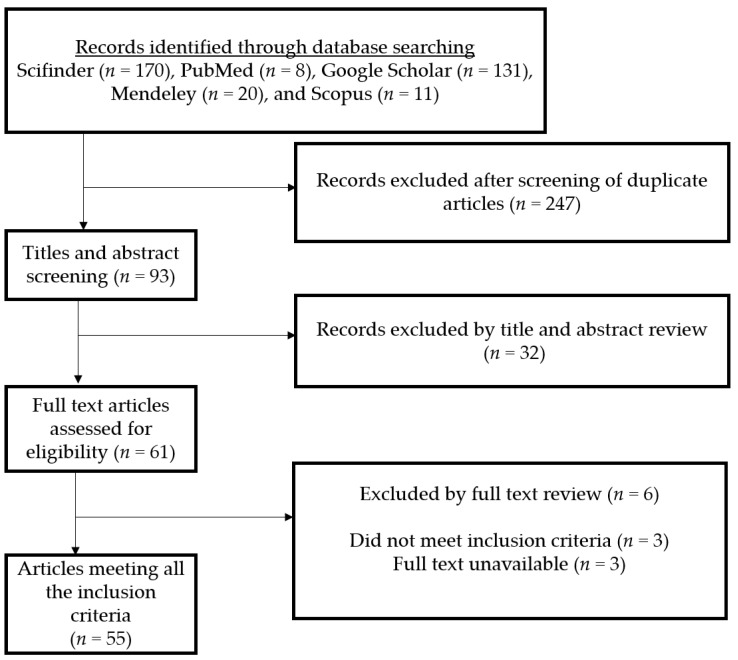
Systematic review and meta-analysis preferred reporting items.

This study was initiated with a literature search related to the *Guarea* genus, and all the synonym names were confirmed based on a plant database “www.theplantlist.org (accessed on 28 August 2022)”. Articles related to the biological and phytochemical properties between 1962 and 2022 were collected from the primary literature research through Scifinder (*n* = 170), PubMed (*n* = 8), Google Scholar (*n* = 131), Mendeley (*n* = 20), and Scopus (*n* = 11) databases and after removing duplicates (*n* = 247), 93 records were identified for title and abstract revision [1,2,3,4,5,6,7,8,9,10,11,12,13,14,15,16,17,18,19,20,21,22,23,24,25,26,27,28,29,30,31,32,33,34,35,36,37,38,39,40,41,42,43,44,45,46,47,48,49,50,51,52,53,54,55,56,57,58,59,60,61,62,63,64,65,66,67,68,69,70,71,72,73,74,75,76,77,78,79,80,81,82,83,84,85,86,87,88,89,90,91,92,93] (Figure 1). Therefore, at the end of the selection process, 61 articles were screened and 32 articles were included in the systematic review (Figure 1).

*Guarea* belongs to the Meliaceae family which is widely distributed in America and Africa. The diameter of this genus is one meter and its tree is usually 20–45m-tall while the characteristics include leave-pinnate, generative reproduction, and 2–8-valved loculicidal fruit. Its staminal tube is 0.4–1.3 cm in length, and the seeds are often shaped like the segment of an orange, with a fleshy, sometimes vascularized, or mealy sarcotesta, and usually thickened on the adaxial surface [35].

## 3. Phytochemistry

### 3.1. Overview of Isolated Compounds Derived from Guarea Genus

**Figure 2:**
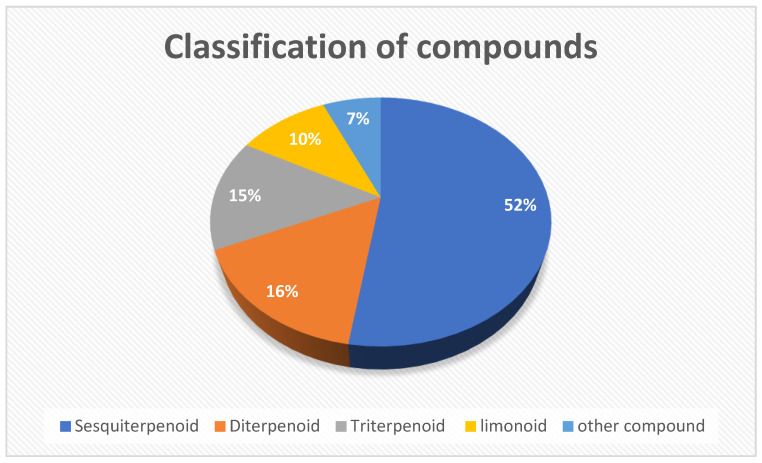
The distribution by groups of compounds from the *Guarea* genus.

About 240 compounds have been isolated from the stembark, leaves, fruits, bark, seed, flowering branches, and root of this genus, based on the literature from 1962 to 2022 as shown in Table 1. The extract for the isolation process was obtained from various solvents such as n-hexane, chloroform, methanol and n-butanol. The first step of the process is the maceration of the dried sample with solvent, especially methanol or ethanol; after that, MeOH/EtOH extract is diluted with water and partitioned with other solvents for obtaining crude extract. Meanwhile, between the hydrodistillation and isolation process is different. The hydrodistillation process used a fresh sample (part of *Guarea*) and submitted to a Clevenger-type apparatus for 4 h for the gained crude oil. The crude extract and crude oil were purified with various techniques such as column chromatography on silica gel or RP-18 silica gel, Sephadex LH-20, preparative TLC, and semipreparative HPLC on RP-18 column for crude extract. The compounds were identified by NMR, mass spectrometry, FTIR, UV, and polarimeter. Moreover, the crude oil was analyzed using a combination of the four techniques of GC, GC/MS, ^1^H-, and ^I3^C-NMR. The compounds identified from the isolation and hydrodistillation processes included 52% sesquiterpenoid, 16% diterpenoid, 15% Triterpenoid, 10% limonoid, as well as 7% non-terpenoid and limonoid. The distribution of the compounds is presented in Figure 2 and the biological activities of the identified compounds are shown in Table 2.

### 3.2. Sesquiterpenoid

**Figure 3:**
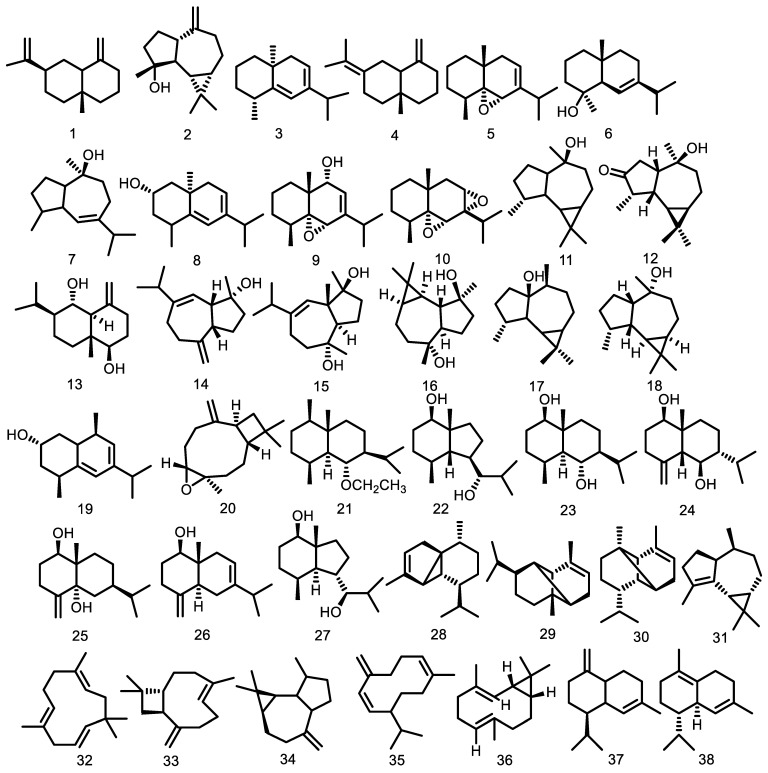

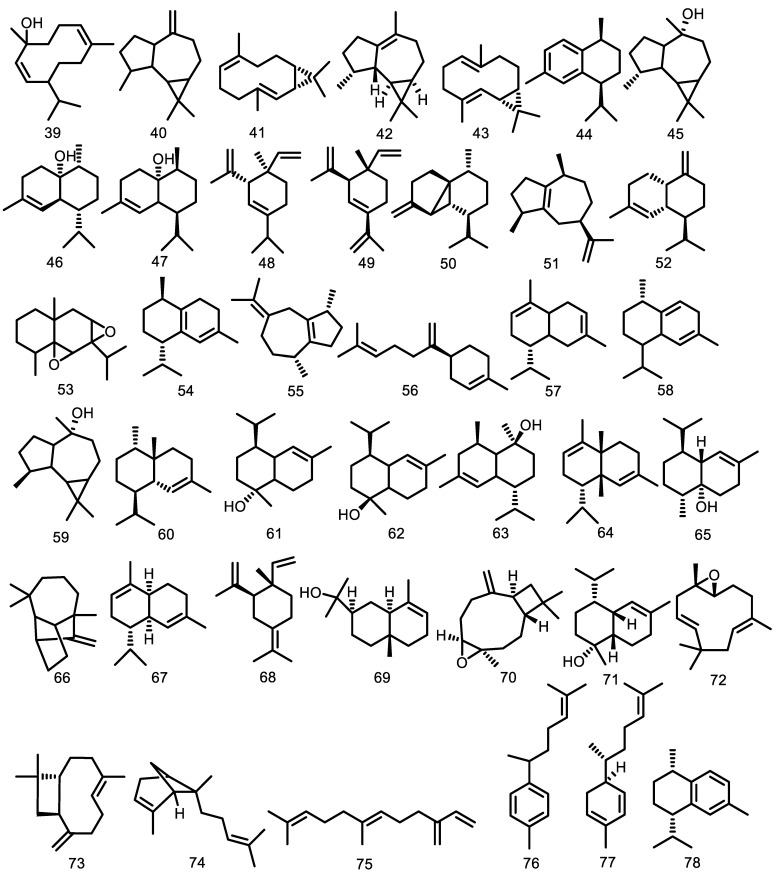

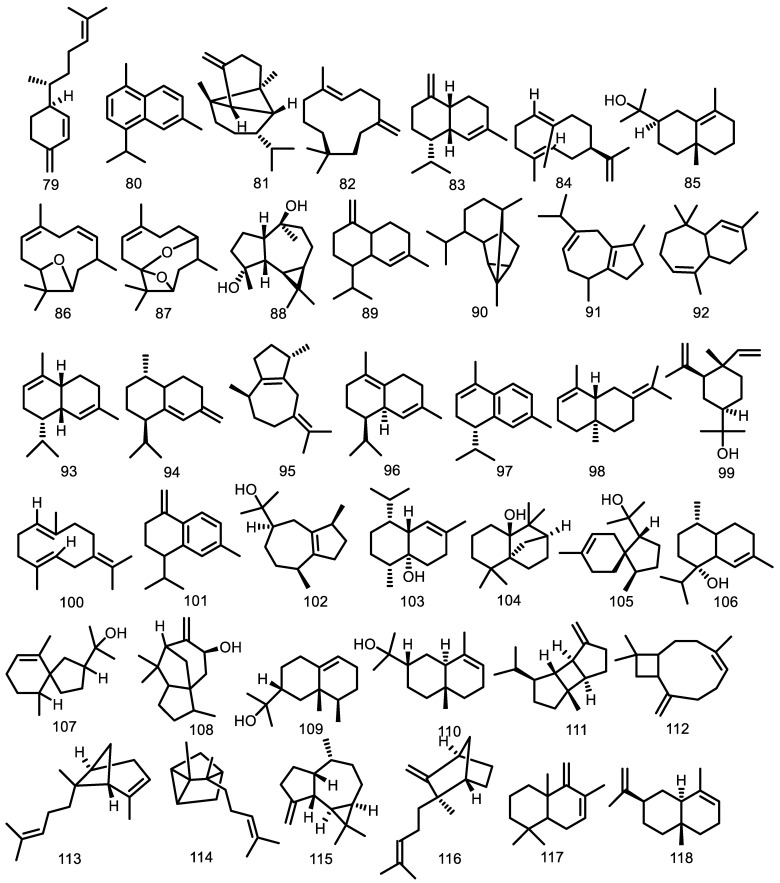

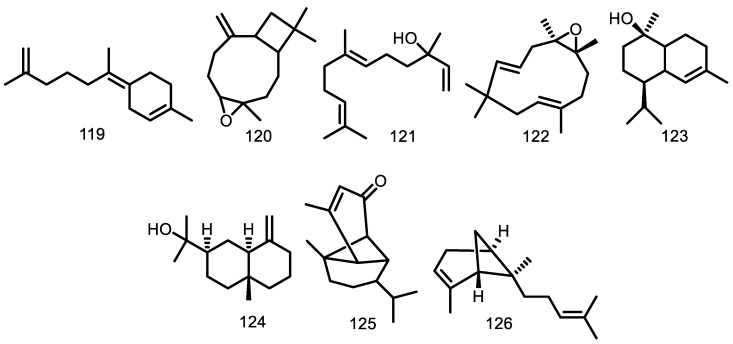
Sesquiterpenoid from *Guarea* species.

About 126 sesquiterpenoids have been isolated from the extract and essential oil since 1995 from *Guarea guidonia*, *G. kunthiana*, *G. thompsonii*, *G. cedrata*, *G. macrophylla*, *G. scabra*, *G. convergens*, and *G. sylvatica*. They include eudesmane, aromadendrane, guaian, caryophyllene, cadinene derivative, opposite, humulene, germacrene, bicyclogermacrene, cadinene, elemene, bisabolene, longifolene, farnasene, cyclosativene, himachalene, isolongifolane, acorenol, hinesol, cedrane, bourbonene, bergamotene, santalene, drimane, mustakone, and eremophilane as indicated in Figure 3.

Cadinene is a significant sesquiterpenoid from the Guarea genus with twenty-eight compounds. Menut et al. [36] reported that the hydrodistillation of essential oil from *G. cedrata* bark produced four compounds of cadinene-type, namely γ-muurolene (**52**), cadina-1,4-diene (**58**), τ-cadinol (**61**), and α-muurolene (**67**). Moreover, the essential oil of *G. macrophylla* has been reported as cadinene-type. About twenty-four compounds were also obtained from leaves, fruits, and stem bark essential oil. Lago and Roque [37] discovered two cadinene types, γ-cadinene (**37**) and δ-cadinene (**38**), isolated from the leaf essential oil of G. macrophylla. In the same year, Lago et al. [38] also obtained cadinene type from the stem bark essential oil of G. macrophylla including (**38**), *cis*-calamenene (**44**), *cis*-cubenol (**46**), and *trans*-cubenol (**47**). The other seven compounds isolated from the hydrodistillation of *G. macrophylla* fruits [39] include cadina-1(6),4-diene (**54**), β-cadinene (**57**), 1-*epi*-cubenol (**60**), τ-cadinol (**61**), τ-muurolol (**62**), α-cadinol (**63**) with four previous cadinene-type compounds. Furthermore, α-cadinene (**64**) and 1-cubenol (**65**) were isolated from the leaf essential oil [40]. Ribeiro et al. [41] also discovered γ-amorphene (**83**) with four previous cadinene type compounds such as (**37**), (**38**), (**52**), (**67**) in 2006. A total of seven other compounds were also obtained from these species such as α-amorphene (**93**), trans-muurola-4(14),5-diene (**94**), δ-amorphene (**96**), α-calacorene (**97**), β-calacorene (**101**), 1,10-di-epi-cubenol (**103**), and cis-cadin-4-en-7-ol (**106**) from the leaf essential oil [42]. Núñez and Roque [43] obtained cadinene from stem bark essential oil and other species of *G. guidonia*. The compounds isolated were trans-4,10(14)-cadinadiene (**89**), (**52**), and (**38**). Six years later, Nunez et al. [44] identified α-muurolol (**71**), (**52**), (**37**), and (**38**) from the branch essential oil. One compound from the leaf essential oil of *G. scabra* was epi-α-cadinol (**123**) [45], and two compounds were isolated from the leaves of *G. kunthiana* calamenene (**78**) and cadalene (**80**) [46].

Eudesmane is the second largest sesquiterpenoid from *Guarea* after the cadinene type with 22 compounds from the hydrodistillation and isolated process. α-eudesmol (**69**) was isolated from the bark essential oil of *G. cedrata*, and the first eudesmane type was reported from this genus [36]. Garcez et al. [47] reported one eudesmane from the wood bark of *G. guidonia*, namely, voleneol (**13**). β-selinene (**1**) was also reported in the leaves and essential oil of *G. guidonia* [48,49]. Furthermore, several compounds were isolated from the leaves such as eudesm-5,7-dien (**3**), eudesm-4,11-diene (**4**), 5α,6α-epoxy-eudesm-7-ene (**5**), eudesm-6-en-4β-ol (**6**), 5α,6α-epoxy-eudesm-7-en-9-ol (**9**), 5α,6α,7α,8α-diepoxy-eudesmane (**10**), and (2S*)-eudesm-5,7-dien-2-ol (**19**) [50]. About five eudesmane compounds were isolated from the seeds of *G. guidonia*, including 6α-ethoxyeudesm-4(15)-en-1β-ol (**21**), eudesm-4(15)-ene-1β,6α-diol (**23**), 5-epi-eudesm-4(15)-ene-1β,6β-diol (**24**), eudesm-4(15)-ene-1β,5α-diol (**25**), and eudesm-4(15),7-dien-1β-ol (**26**) [51]. In addition, 5,6,7,8-diepoxy-eudesmane (**53**) and eudesm-5,7-dien-2α-ol (**8**) were obtained from leaf essential oil [49]. Ribeiro et al. [41] isolated γ-eudesmol (**85**) from the leaf essential oil of *G. macrophylla*, while Oliveira et al. [42] reported two compounds, namely selina-3,7(11)-diene (**98**) and 7-epi-α-eudesmol (**110**). Two eudesmane types, α-selinene (**118**) and β-eudesmol (**124**), were also isolated from branch essential oil of *G. convergens* and *G. silvatica* [45].

Furthermore, aromadendrane types such as allo-aromadendrene (**34**), viridiflorene (**42**), globulol (**45**), and epi-globulol (**59**) were obtained from the bark essential oil of *G. cedrata* [36]. Other species, such as *G. macrophylla*, *G. guidonia*, *G. kunthiana*, were found to also contain similar compounds. Spathulenol (**2**) and palustrol (**17**) were first isolated from the leaves of *G. macrophylla* [52] while essential oil from the leaves and the stem bark were also reported to contain aromadendrane type. Lago et al. [37] isolated ledol (**18**), and α-gurjunene (**31**) from the leaves and aromadendrene (**40**) from stem bark essential oil [38]. Seven years later, alloaromadendrane-4α,10β-diol (**88**) was isolated from the bark [53]. Two aromadendrane types, viridiflorol (**11**) and 3-oxo-10-alloaromadendranol (**12**), were also obtained from the wood bark of *G. guidonia* [47], (-)-4β,10α-aromadendranediol (**16**) from the leaves of *G. kunthiana* [54], and β-gurjunene (**115**) from *G. scabra* [45].

Furthermore, guai-6-en-10β-ol (**7**) was the first guaian type isolated from the leaves of *G. macrophylla* [52]. Compounds such as cis-*β*-guaiene (**55**), 6,9-guaiadiene (**91**), trans-*β*-guaiene (**95**), and guaiol (**102**) were isolated from the fruit and leaf essential oil [39,42]. *G. kunthiana* also has a guaian type, while alismol (**14**) and alismoxide (**15**) were identified from the leaves [54]. In addition, α-guaiene (**51**) was obtained from the leaf essential oil of *G. guidonia* [49].

Caryophyllene oxide (**20**) and β-caryophyllene (**33**) were identified from the bark essential oil of *G. cedrata* [36]. Núñez and Roque [43] reported isocaryophyllene oxide (**70**) from the stem bark essential oil of *G. guidonia*. Meanwhile, two other species, *G. kunthiana* and *G. macrophylla*, were found to contain E-caryophyllene (**73**) and 9-epi-β-caryophyllene (**82**) [41,46]. Magalhães et al. [45] also reported two compounds, *cis*-caryophyllene (**112**) and caryophyllene epoxide (**120**), from the leaf essential oil of *G. scabra* and branches of *G. humatensis*.

The derivative compounds from the cadinene type, such as α-cubebene (**28**) and β-copaene (**81**), were obtained from the leaf and stem bark essential oil of *G. macrophylla* [37,38,41]. Furthermore, α-ylangene (**29**) and α-copaene (**30**) were first identified from the bark essential oil of *G. cedrata* [36], while *G. guidonia* was found to contain β-cubebene (**50**) [49].

The α-humulene (**32**) and 6,7-epoxy-2,9-humuladiene (**72**) humulene type were identified from the stem bark essential oil of *G. guidonia* [43]. Furthermore, 1(10)-epoxy-4,7-humuladiene (**86**) and 1(10),4-diepoxy-7-humulene were also obtained from the bark (**87**) [47]. The latest discovery was performed by Magalhães et al. [45], where one humulene-type sesquiterpenoid humulene epoxide II (**122**) was identified from the branch essential oil of *G. silvatica*.

Nunez and Roque [43] identified germacrene D (**35**) from the stem bark essential oil of *G. guidonia*, while the *G. macrophylla* species was found to contain germacrene-D-4-ol (**39**), germacrene A (**84**), and germacrene B (**100**) in the leaf essential oil [37,41,42]. Moreover, bicyclogermacrene type was also identified from the leaf and stem bark essential oil of *G. macrophylla* including bicyclogermacrene (**36**), cis-bicyclogermacradiene (**41**), and *trans*-bicyclogermacradiene (**43**) [37,38].

The bark essential oil from *G. cedrata* was reported to contain elemene-type sesquiterpenoid γ-elemene (**68**) [36]. β-elemene (**49**) was also isolated [43] from the stem bark essential oil of *G. guidonia*. In 2005, δ-elemene (**48**) was reported in the leaf essential oil of this species [49], while elemol (**99**) was identified in the leaf essential oil of *G. macrophylla* [42].

Eight compounds with bisabolene-type sesquiterpenoids were obtained from four species, namely *G. macrophylla*, *G. kunthiana*, *G. sylvatica*, and *G. scabra*. β-bisabolene (**56**) was obtained from the fruit essential oil of *G. macrophylla* [39]. Magalhães et al. [45] also identified three compounds, namely (E)-iso-γ-bisabolene (**119**) from the branch essential oil of *G. silvatica*, as well as α-*cis*-bergamotene (**113**) and α-*trans*-bergamotene (**126**) from the leaf essential oil of *G. scabra*. Eight years later, α-bergamotene (**74**), α-curcumene (**76**), α-zingiberene (**77**), and β-sesquiphellandrene (**79**) were isolated from the leaf essential oil of *G. kunthiana* [46].

Furthermore, minor-type sesquiterpenoids were obtained from this genus, such as two compounds of opposite-type sesquiterpenoid (7R*)-5-epi-opposit-4(15)-ene-1β,7-diol (**22**) and (7R*)-opposit-4(15)-ene-1β,7-diol (**27**) from the seeds of *G. guidonia* [51], while longifolene (**66**) was isolated from the bark essential oil of *G. cedrata* [36]. Two compounds of acyclic sesquiterpenoids, β-farnesene (**75**) and *trans*-nerolidol (**121**), were identified from the leaf essential oil of *G. kunthiana* and *G. scabra* [45,46]. Moreover, cyclosativene (**90**), γ-himachalene (**92**), isolongifolan-7-α-ol (**104**), α-acorenol (**105**), hinesol (**107**), cedr-8(15)-en-9α-ol (**108**), and valerianol (**109**) were isolated from the leaf essential oil of *G. macrophylla* [42]. Magalhães et al. [45] also reported five other compounds, such as β-bourbonene (**111**) from the leaf essential oil of *G. scabra*; α-santalene (**114**), β-santalene (**116**), drima-7,9(11)-diene (**117**) from the branches of *G. convergens;* and mustakone (**125**) from *G. silvatica*. All the sesquiterpenoid structures are shown in Figure 2.

### 3.3. Diterpenoid

Diterpenoid of 16% was isolated from the *Guarea* genus with two major types, isopimarane and labdane. One of the diterpenoid types which was first reported by Lago et al. was isopimarane [52] from the leaves of *G. macrophylla* with three types, namely isopimara-7,15-dien-3-one (**150**), isopimara-7,15-dien-3β-ol (**132**), and isopimara-7,15-dien-2β-ol (**151**). Afterward, five diterpenoids, namely, 7α-hydroperoxy-isopimara-8(14),15-diene-2α,3β-diol (**148**), 19-nor-isopimara-7,15,4(18)-trien-3-one (**149**), isopimara-7,15-dien-2α-ol (**152**), isopimara-7,15-diene (**158**), and isopimara-7,15-diene-2α,3β-diol (**131**), were isolated and identified from the leaf essential oil of *Guarea macrophylla* from [37,55,56].

Four types of labdane diterpenoids, namely, 3-oxo-labd-8(17),12Z,14-triene (**133**), 3α-hydroxylabd-8(17),12Z,14-triene (**134**), 3β-hydroxylabd-8(17),12Z,14-triene (**135**), and 19-hydroxymanoyloxide (**135**)—identified from the leaves of *G. trichilioides*—were reported in 1996 by Furlan et al. [57]. Furthermore, three labdane-type compounds such as manoyl oxide (**153**), labda-8,14-dien-13-ol (**154**), and labda-8,13-(E)-dien-15-ol (**159**), were isolated from the leaves of *G. macrophylla* [52], while *ent*-13-epimanoyloxide (**147**) was obtained from the leaves of *G. kunthiana* [54].

Cneorubin A (**111**), B (**112**), X (**113**), and Y (**114**) were isolated from the leaves and the aerial parts of *G. guidonia* [48,58], while three kaurene types of diterpenoid compounds, *ent*-kaur-16-en-2-one (**139**), *ent*-kaur-16-ene (**140**), and *ent*-3β- and 3α-hydroxykaur-16-ene (**141** and **142**), were obtained from the leaves of *G. kunthiana* [54]. Additionally, Magalhães et al. [45] identified kaurene (**164**) from the leaf essential oil of *G. sylvatica*.

Diterpenoids of the sandaracopimaradeine type were identified in the leaves of *G. rhophalocarpa*. The compounds were *ent*-8(14),15-sandaracopimaradiene-2α,18-diol (**156**), and *ent*-8(14),15-sandaracopimaradine-2β,18-diol (**157**) [59]. Eighteen years later, sandaracopimarinal (**163**) was identified from the leaf essential oil of *G. macrophylla* [42].

Furthermore, two diterpenoids of the clerodane type, (-)-2-oxo-13-hydroxy,3,14-clerodandiene (**136**) and 13-hydroxy-3,14-clerodandiene (**138**), were obtained from the leaves of *G. trichilioides* [57]. An investigation to identify three other compounds, including kolavelool (**143**), kolavenol (**144**), and kolavenal (**145**) from the leaves of *G. kunthiana*, was conducted by Garcez et al. [54].

**Figure 4:**
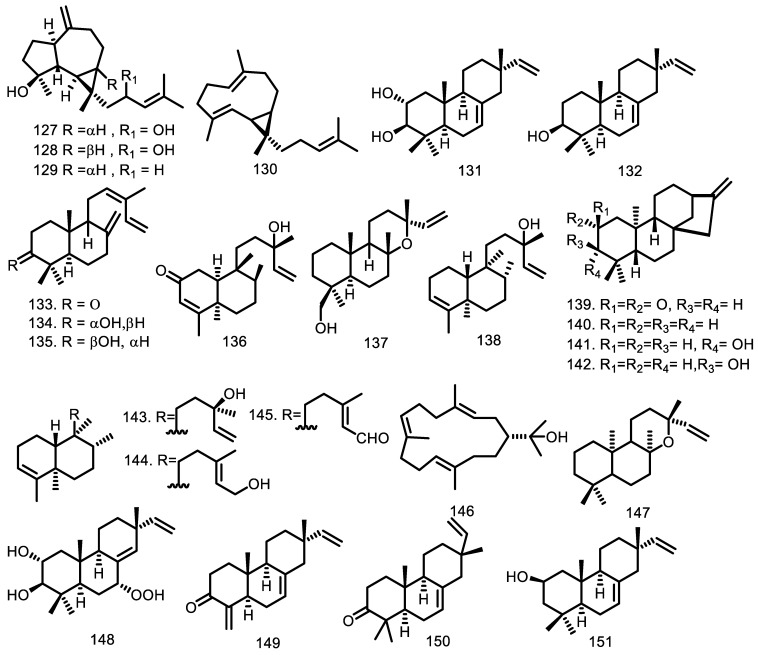

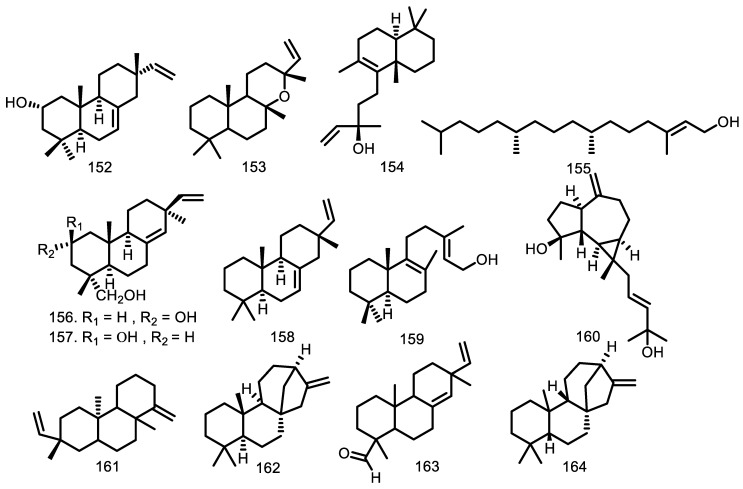
Diterpenoid from *Guarea* species.

The acyclic type, phytol (**155**), was identified from the leaves of *G. macrophylla* and *G. guidonia* [55,60]. Garcez et al. [54] isolated (-)-nephthenol (**146**) from the leaves of *G. kunthiana*, while one prenylaromadendrane-type boscartol C (**160**) was obtained from the aerial parts of *G. guidonia* [58]. One of the dolabradiene types, 13-epi-dolabradiene (**145**), was identified from the leaf essential oil of *G. macrophylla*, along with phyllocladane (**146**) [42]. The diterpenoid structures are presented in detail in Figure 4.

### 3.4. Triterpenoid

**Figure 5:**
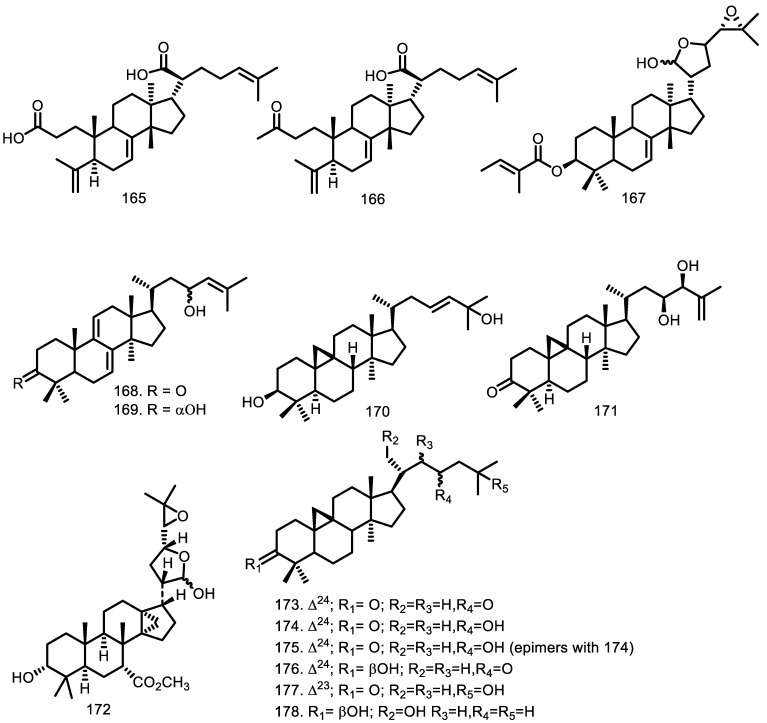

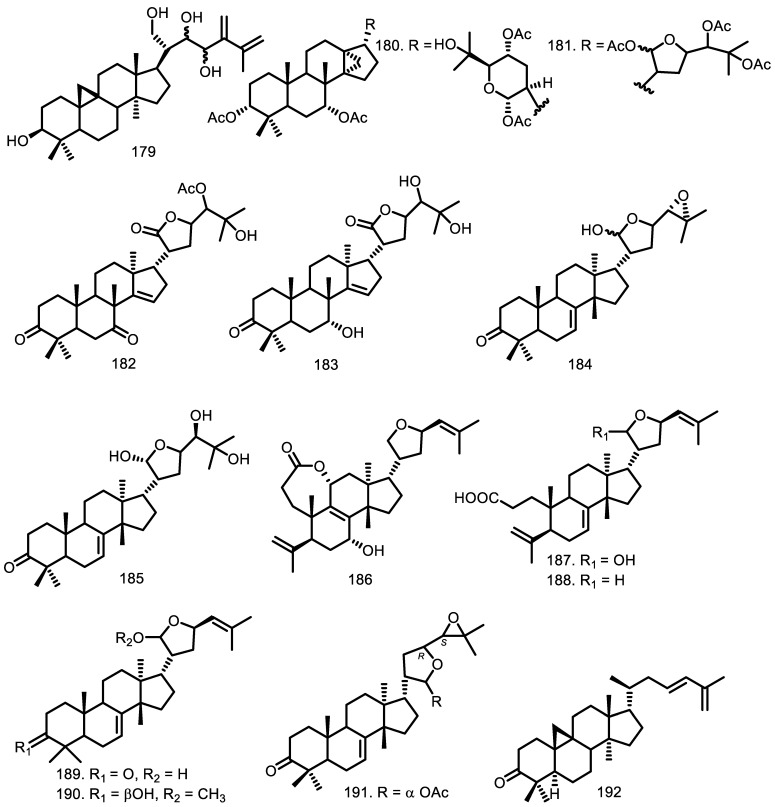

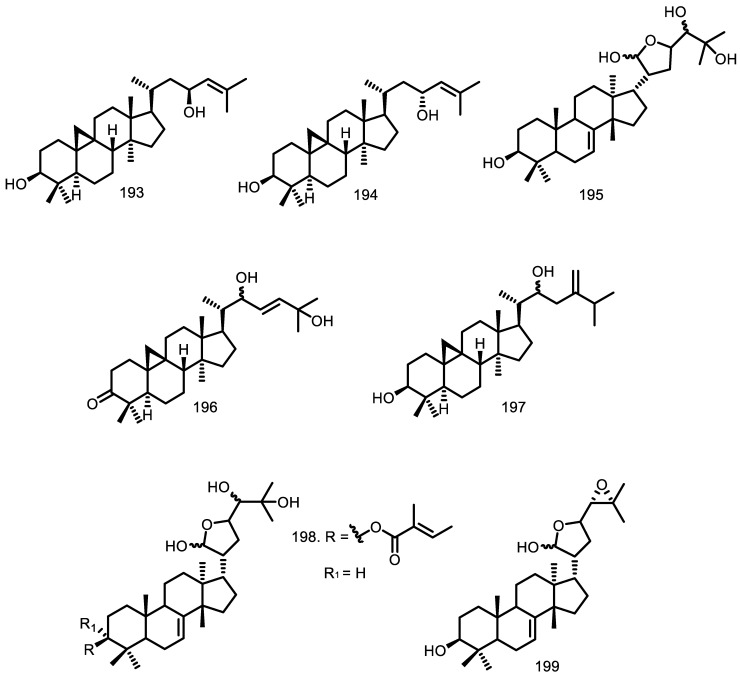
Triterpenoid from *Guarea* species.

Thirty-five compounds were identified as triterpenoids, such as tirucallane, protolimonoid, lanostane, cycloartane, glabretal, glabretal derivatives, and apotirucallane (Figure 5). Cycloartane was the major triterpenoid type isolated from the *Guarea* genus. In 1993, seven compounds (cycloart-24-en-3,23-dione (**173**), 23-hydroxycycloart-24-en-3-one (epimers) (**174** and **175**), 3β-hydroxycycloart-24-en-23-one (**176**), 25-hydroxycycloart-23-en-3-one (**177**), 3β-21-dihydroxycycloartane (**178**), and 3β,21,22,23-tetrahydroxycycloartane-24(31), 25-diene (**179**)) were identified from the leaves of *G. trichilioides* [61]. Furthermore, 22,25-dihydroxycycloart-23*E*-en-3-one (**196**), 24-methylenecycloartane-3β,22-diol (**197**), and cycloarta-23,25-dien-3-one (**192**) were obtained from the leaves of *G. macrophylla* [52,62], while two cyloartanes, namely (23S*)-cycloart-24-ene-3β,23-diol (**193**) and (23R*)-cycloart-24-ene-3β,23-diol (**194**), were isolated from the leaves of *G. guidonia* [60]. In the same year, cycloart-23*E*-ene-3β,25-diol (**170**) was discovered in the leaves of *G. macrophylla* [62], while in 2017, Conserva et al. [56] obtained (23S*,24S*)-dihydroxycicloart-25-en-3-one (**171**).

Two lanostane-type compounds, 23-hydroxy-5α-lanosta 7,9(11),24-triene-3-one (**168**) and 5α-lanosta-7,9(11),24-triene-3α,23-diol (**169**), were obtained from the leaves of *Guarea rhophalocarpa* [59], while glabretal (**172**) was identified from heartwood of *G. glabra*. Furthermore, 21,24-epoxy-3α,7α,21,23-tetraacetoxy-25-hydroxy-4α,4β,8β-trimethyl-14,18-cyclo-5α,13α,14α,17α-cholestane (**181**), and 21,23-epoxy-3α,7α,21,24,25-pentaacetoxy-4α, 4β,8β-trimethyl-14,18-cyclo-5α,13α,14α,17α-cholestane (**182**) as glabretal derivatives were identified from the leaves and twigs of *G. jamicensis* [63,64].

The 3,4-secotirucalla-4(28),7,24-trien-3,21-dioic acid (**165**) and 3,4-secotirucalla-4(28),7,24-trien-3,21-dioic acid 3-methyl ester (**166**) as tirucallane types of triterpenoid were reported by Akinniyi et al. [33] from the bark of *G. cedrata*. Furthermore, four tirucallane types, guareolide (**186**), guareoic acid A (**187**) and B (**188**), flindissone (**189**), as well as picroquassin E (**190**), were isolated from the aerial parts of *G. guidonia* [58].

Jimenez et al. [65] reported that three protolimonoid types, melianone (**184**), melianodiol (**185**), and 21-α-acetylmelianone (**191**), were first isolated from the seeds of *G. grandiflora*. In 2015, four compounds of this type were also identified, including 3β-*O*-tigloylmelianol (**167**), 3β-*O*-tigloylmeliantriol (**198**), and melianol (**199**), from the fruits of *G. kunthiana* [66]. Moreover, 24-acetoxy-25-hydroxy-3,7-dioxoapotirucalla-14-en-21,23-olide (**182**) and 7α,24,25-trihydroxy-3-oxoapotirucalla-14-en-21,23-olide (**183**) as apotirucallane types were isolated from the leaves and branches of *G. convergens* [67].

### 3.5. Limonoid

Limonoids are classified into many classes based on the type of skeleton [68,69], and about eleven classes have been reported from this genus. The first exploration by Housley et al. [34] reported dihydrogedunin (**221**) from the heartwood of *G. thompsonii*.

Connollyl et al. [70] also found one andirobine-type limonoid, namely methyl 6-acetoxyangolensate (**206**), identified from the bark of *G. thompsonii* and methyl angolensate (**214**) from the fruits of *G. kunthiana* [70,71]. Moreover, one of limonoid types which was called with dregeanin (**207**) was obtained from the bark of *G. thompsonii*, and rohituka-type named with 2’-hydroxyrohitukin (**215**) was identified from the bark of *G. cedrata*. The obakunol-type limonoid, 7-acetyldihydronomilin (**216**), was isolated from the aerial parts of *G. guidonia*, and the ecuadorin (**217**) which was one of the ecuadorin-types, was found in the aerial parts of *G. kunthiana* [33,58,70,72].

Prieurianin (**219**) and 14,15β-epoxyprieuriani (**210**) were found in the root bark of *G. guidonia* as a prieurianin-type limonoid [73]. Garcez et al. [47] also reported mombasol (**208**) from the bark of *G. guidonia* and the investigation by Lukacova et al. [73] obtained 7-oxo-gedunin (**218**) from the root bark, while three gedunin limonoids, 7-deacetoxy-7-oxogedunin (**200**), gedunin (**201**), and 6α-acetoxygedunin (**209**), were isolated from the seeds of *G. grandiflora* [65].

Zelnik and Rosito [74] discovered one mexicanolide type, called fissinolide (**220**), in the seeds of *G. trichilioides*. Five years later, the seeds were found to also contain angustinolide (**224**) [75]. Humilinolide E (**211**), methyl 2-hydroxy-3b-tigloyloxy-1-oxomeliac-8(30)-enate (**212**), and swietenine acetate (**213**) were isolated from the fruits of *G. kunthiana* [71]. Furthermore, an investigation by Bellone et al. [76] identified 3-(2′-hydroxyisovaleroyl) khasenegasin I (**205**) from the stem bark of *G. guidonia*.

**Figure 6:**
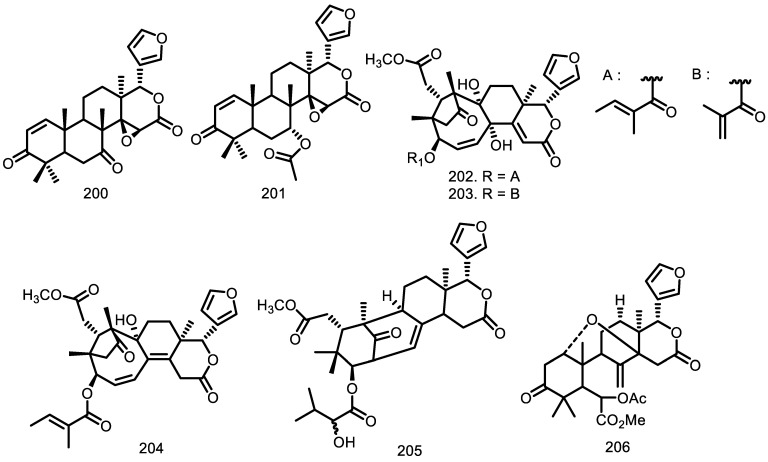

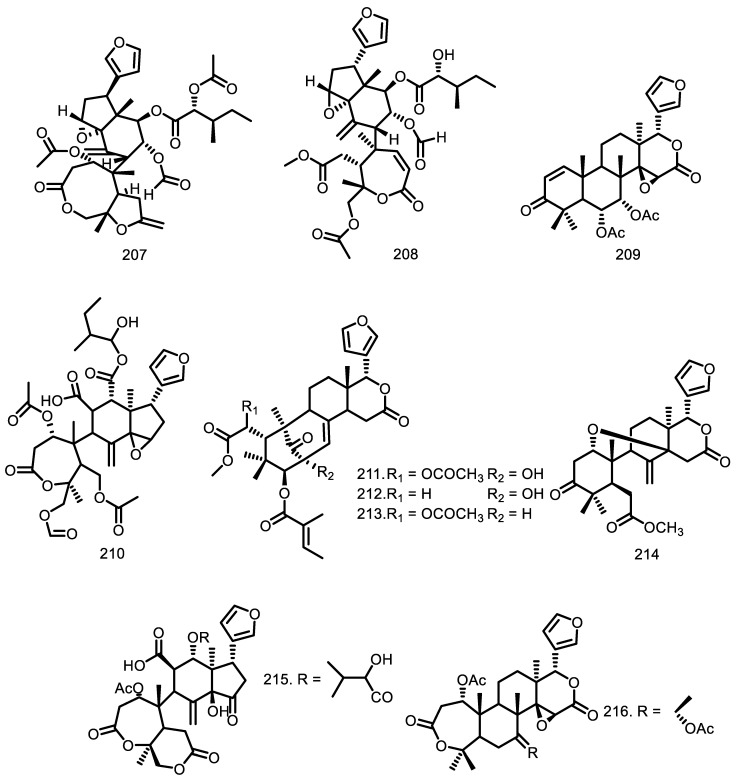

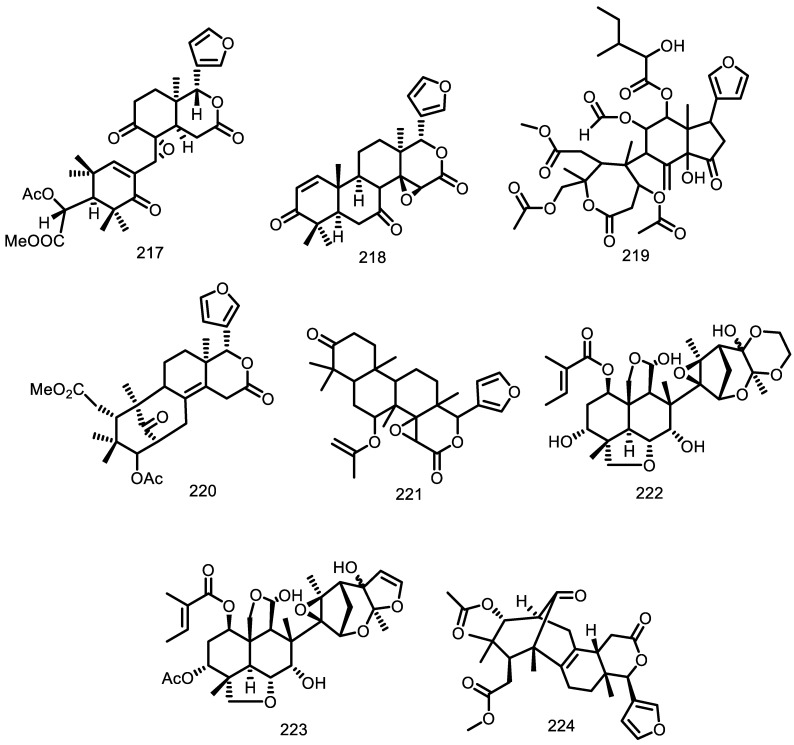
Limonoid from *Guarea* species.

The twigs of *G. mayombensis* produced azadirachtin-type mayombensin (**222**) and azadirachtin I (**223**) [77]. Meanwhile, three compounds of A2, B, D-seco skeletons such as chisomicine D (**202**), chisomicine E (**203**), and chisomicine F (**204**), were identified from the stem bark of *G. guidonia* [76] (Figure 6).

### 3.6. Steroid

**Figure 7:**
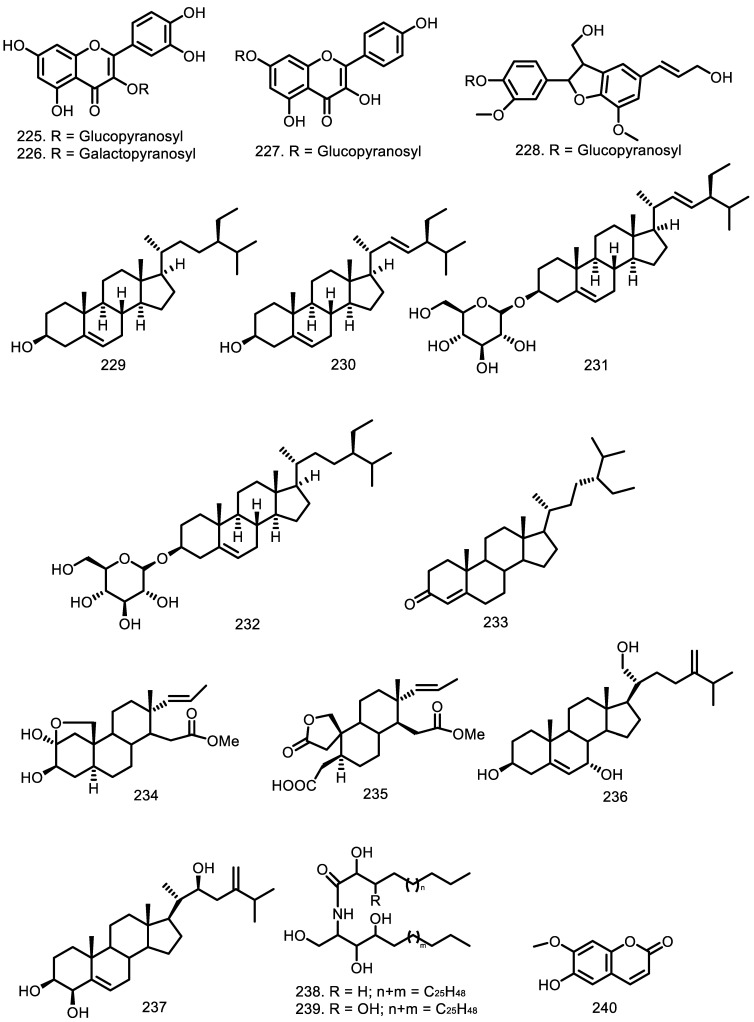
Other compounds from *Guarea* species.

Ergostane- and pregnane-type steroids were isolated from the *Guarea* genus, along with general steroid compounds such as β-sitosterol (**229**), stigmasterol (**230**), and β-sitostenone (**233**) [48,67,78]. Furthermore, the steroids glycoside stigmasterol glucoside (**231**) and β-sitosterol glucoside (**232**) were obtained from the twigs of *G. mayombensis* [77], while two ergostane-type steroids, ergosta-5,24(24′)-diene-3β,7α,21-triol (**236**) and ergosta-5,24(24′)-diene-3β,4β,22S-triol (**237**), were identified from the leaves and branches of *G. convergens* [67]. Garcez et al. [79] also reported two pregnane-type steroids, 2α,3β-dihydroxy-16,17-seco-pregn-17-ene-16-oic acid methyl ester 2β,19-hemiketal (**234**) and 2,3:16,17-di-seco-pregn-17-ene-3-oic acid-16-oic acid methyl ester-19-hydroxy-2-carboxylic acid-2,19-lactone (**235**), from the trunk bark of *G. guidonia* (Figure 7).

### 3.7. Other Compounds

Flavonoid, lignan, ceramide, and coumarin were also identified from this plant genus. Quercetin 3-*O*-β-d-glucopyranoside (**225**), quercetin 3-*O*-β-d-galactopyranoside (**226**), and kaempferol 7-*O*-β-d-glucopyranoside (**227**) as glucoside flavonoids were isolated from the flowering branches of *G. macrophylla*. Furthermore, one neolignane compound, dehydrodiconiferyl alcohol-4-β-d-glucoside (**228**), was reported from the same part of this species [80]. Two ceramides, ceramide A (**238**) and B (**239**), were obtained from the twigs of *G. mayombensis* [77], while one coumarin, scopoletin, (**240**) was found in the leaves of *G. rhopalocarpa* [59] (Figure 7).

## 4. *Guarea* Bioactivity

Plants of the genus *Guarea* have long been used in traditional medicine in several countries for relieving body aches, diarrhea, angina, asthma, and dyspnea. The boiled leaves are used as an emetic [81]. Several biological tests conducted showed that the plant extract has cytotoxic, antimalarial, anti-inflammatory, antimicrobial, insecticidal, antioxidant, antiparasitic, antiprotozoal, antiviral, and phosphorylation inhibitor activities [58,59,82,83,84,85,86,87,88,89] (Table 2).

### 4.1. Cytotoxic

The cytotoxic activity of the Guarea genus has been studied in many extracts and compounds (diterpenoids, triterpenoids, limonoids, and steroids) using various test methods. The findings could lead to the development of new antitumor and anticancer drugs. The extract and the compounds of four species from the *Guarea* genus were evaluated in 1962. Lukacova et al. [73] identified three compounds from *G. guidonia*, including 14,15β-epoxyprieuriani (**210**), 7-oxo-gedunin (**218**), and prieurianin (**219**). The compounds **210** and **219** are active against the leukemia cell line P388 ED_50_ 0.47–0.74 µg/mL and P388 ED50 4.4–7.8 µg/mL, respectively, while **218** is not active. Furthermore, methylene chloride extract was evaluated against U-937 cell lines; bark and leaf extract of *G. polymera* each showed a lethal dose (LD_50_) of 6.1 ± 0.5 µg/mL and 6.1 ± 1.2 µg/mL while the seed of *G. guidonia* had a LD_50_ of 28.8 ± 8.2 µg/mL [90].

The six compounds from *G. rhophalacarpa ent*-8(14), namely 15-sandaracopimaradiene-2α,18-diol (**156**), *ent*-8(14),15-sandaracopimaradine-2β,18-diol (**157**), 23-hydroxy-5α-lanosta 7,9(11),24-triene-3-one (**168**), 5α-lanosta-7,9(11),24-triene-3α,23-diol (**169**), stigmasterol (**230**), and scopoletin (**240**), were tested against the KB cell line with an inhibitory concentration (IC_50_) of 48 µM, 75.8 µM, 30.2 µM, 21.2 µM, > 1272 µM, and 130.2 µM, respectively [59].

Four compounds from *G. macrophylla* were also tested against the five cancer cell types B16F10-Nex2, A2058, MCF-7, HL-60, and HeLa. Cycloart-23E-ene-3β,25-diol (**170**) had the best activity compared to the other three compounds. Meanwhile, the results of the tests against HL-60, HeLa, B16F10-Nex2, A2058, and MCF-7 were 18.3, 52.1, 58.9, 60.7 and 63.5 µM, respectively. Two other compounds, isopimara-7,15-dien-2α,3β-diol (**131**) and isopimara-7,15-dien-3β-ol (**132**), have activity over 100 µM against five cell lines [56].

Hernandez et al. [58] identified five compounds of which three have an EC_50_ under 100 µM. Five compounds were also tested against the Jurkat, HeLa, MCF-7, and PBMC cell lines. Flindissone (**189**) showed activity with EC_50_ 25, 27, 50, and > 100 µM for the Jurkat, HeLa, MCF-7, and PBMC cell lines, while guareoic acid A (**187**) had a high EC_50_ against the Jurkat cell line with a value of 39 µM. Moreover, picroquassin E (**190**), guareolide (**186**), and guareoic acid A (**187**) showed no activity against PBMC (nontumor human peripheral blood mononuclear cell line).

In a recent cytotoxic assay studied by Bellone et al. [76] on four compounds isolated from *G. guidonia*, chisomicine D (**202**) showed inhibitory growth value to U-937 and HeLa cell lines with an IC_50_ 20 ± 3 µM and > 50 µM, but no activity was found against PBMC. Other compounds (chisomicine E (**203**), chisomicine F (**204**), and 3-(2′-hydroxyisovaleroyl) khasenegasin I (**205**)) were also found to be inactive against U-937 and HeLa cell lines.

### 4.2. Anti-Inflamation

Catabolism takes precedence over anabolism in an inflammatory state. It is also a defense mechanism that aids in the elimination of potentially harmful factors and maintains body homeostasis. Because of the increased permeability of capillaries and white blood cells, this causes increased blood flow to the site of inflammation, resulting in symptoms such as redness, swelling, and pain.

Oga et al. [82] reported the anti-inflammation activity from ethanol extract of *G. guidonia* seeds against male Wistar rats. About an 8.0 mL/kg extract dose provided significant inhibition of carrageenin-induced edema, and the effects increased periodically. Similarly, a 5.0 mL/kg extract dose provided effects amounting to 15% on granuloma tissue formation after 2, 4, and 6 days.

### 4.3. Antimalarial

Four extracts from *G. multiflora* were obtained using petroleum ether, methanol, water, and chloroform. They were collected from leaves, stem bark, and wood, as well as fruits. The extracts showed no significant results as three, namely, petroleum ether from leaves, methanol of stem bark and fruits, as well as chloroform from stem bark, had an IC50 of 50 µg/mL. Meanwhile, other extracts showed an IC_50_ of 500 µg/mL and were not active [83].

### 4.4. Antiprotozoal

Chloroform extract from leaves of *G. rhopalocarpa* showed high activity against *Leishmania donovani* with IC_50_ 45 µg/mL. Moreover, methanol and butanol extracts have IC_50_ 62.5 µg/mL and 300 µg/mL, while the water extract has the lowest activity. *Ent*-8(14),15-sandaracopimaradiene-2α,18-diol (**156**) was more active than *ent*-8(14),15-sandaracopimaradine-2β,18-diol (**157**) against *L. donovani* promastigotes with IC_50_ of 16,8 and 49.7 µg/mL, respectively. A study on two triterpenoids showed that 23-hydroxy-5α-lanosta 7,9(11),24-triene-3-one (**168**) is more active than 5α-lanosta-7,9(11),24-triene-3α,23-diol (**169**), tested using *L. donovani* with an IC_50_ of 7.2 µg/mL [59].

Furthermore, Weniger et al. [90] identified methylene chloride extract of bark and leaves of *G. polymera* which has a selectivity index against *Leishmania Viannia panamensis* with a lethal dose/effective dose (LD_50_/ED_50_) of 1.5 µg/mL. The seeds of *G. guidonia* were also active against *Plasmodium falciparum* with an LD_50_/IC_50_ 2.9 µg/mL. Hexane extract obtained from the root of *G. kunthiana* reportedly had antileishmanial activity on the intracellular parasite, *Leishmania donovani*. The test was evaluated using the colorimetric method which was an MTT assay and the extract showed an IC_50_ of 7.9 ± 1.3 µg/mL [84]. Moreover, the 3β-*O*-tigloylmelianol (**167**) was investigated with larvicide and ecydysis tests against the cattle tick of Rhipicephalus (Boophilus) microplus (Canestrini) (Acari: Ixodidae); the compound showed a significant reduction in the number of oocytes [91].

### 4.5. Antiviral

Two water extracts from the fruits and leaves of *G. guidonia* were identified to have antiviral activity against pseudorabies and mouth disease virus in the IB-RS-2 pig cell lines and against bovine herpesvirus 1 (BHV-1) in the GBK bovine cell line. The result of the fruit extract test was more active than the leaves in the IB-RS-2 cell. Meanwhile, the activity of the two extracts increased with an IC_50_ of 62.5 and 125 µg/mL in the GBK cell [85].

### 4.6. Antimicrobial

Several compounds isolated from *Guarea* have been found to have antimicrobial activity. This activity provides antibiotics against microorganisms that can cause food defects, such as pathogens. A study conducted by Pandini et al. reported the result of antimicrobial activity for essential oil and methanol extracts from *G. kunthiana* [88]. Methanol extract showed no activity in the MIC or MBC test. Meanwhile, the essential oil evaluated with MIC and MBC against *S. infantris*, *S. tyrphimurium* and *S. give* showed antimicrobial activity amounting to 54.6 µg/mL. The ethyl acetate extract had activity ranging from 100 to 200 µg/mL.

### 4.7. Insecticidal Activity

Four compounds were isolated from *G. grandiflora* and evaluated against the growth of larva ECB (European corn borer). The results showed that 21-α-acetylmelianone (**191**) and melianone (**184**) have the activity to inhibit the growth of ECB larvae using the fed control diet. Meanwhile, the pupal weight was not affected by any of the compounds but the percentage of pupation was significantly reduced by melianodiol (**185**) [65].

The 10% alcoholic extract from *G. kunthiana* produced the highest percentage of larval mortality, while the 10% aqueous extract exhibited 14.6%. Moreover, 200 mg/mL of essential oil affected 28.6% of larval mortality [88]. The ethyl acetate extract from *G. kunthiana* was also evaluated against *Aedes aegyptyi* with LC_50_ and LC_90_ values of 105.7 µg/mL and 408, 9 µg/mL, respectively. Melianodiol (**185**) exhibited the highest activity with LC_50_ 14.4 and LC_90_ 17.54 µg/mL, while meliantriol (**195**) showed the activity of over 100 µg/mL [87].

### 4.8. Antioxidant and Phosphorylation Inhibitor

The antioxidant activity is a defense mechanism that protects our bodies from oxidative stress caused by free radicals and reactive oxygen species (ROS). Oxidative stress can occur as a result of ROS formation and the detoxification of elevated levels of ROS, resulting in impaired cellular function. The compounds which have been isolated from this genus have antioxidant activity [88]. The essential oil, alcoholic, aqueous, and ethyl acetate ex-tracts were evaluated. Based on the results, the alcoholic extract showed an IC_50_ of 15.3 µg/mL while ethyl acetate had the lowest activity with an IC_50_ 176.8 µg/mL.

On the other hand, two compounds, 7-deacetoxy-7-oxogedunin (**200**) and Gedunin (**201**), which were obtained from G. grandiflora, showed 7-deacetoxy-7-oxogedunin up to 350 µM and could inhibit ATP synthase coupled to electron transfer, while the activity of Mg^2+^-ATPase was only slightly inhibited. Meanwhile, the increased concentration of 7-deacetoxy-7-oxogedunin up to 300 µM did not significantly inhibit the ATP hydrolysis process but ATPase activity caused inhibition of 7 and 6% for Mg^2+^ and Ca^2+^. Gedunin did not significantly inhibit Ca^2+^- and Mg^2+^-dependent ATPase activities [89].

## 5. Conclusions

*Guarea* is one of the largest genera of the Meliaceae family, and about 240 compounds have been obtained through the hydrodistillation and isolation process with the majority of them being sesquiterpenoids. Furthermore, the bioactivity data show that this plant has a variety of activities, specifically for cytotoxic activity.

## Figures and Tables

**Table 1:** Terpenoid and other compounds from *Guarea* Genus.

Compounds	Species	Sources	References
Sesquiterpenoid			
β-selinene (**1**)	*G. guidonia*	Leaves Leaf essential oil	[48,60] [49,50]
Spathulenol (**2**)	*G. guidonia* *G. kunthiana* *G. macrophylla*	Leaves Leaves Leaf essential oil Leaves Wood Leaf essential oil Fruit essential oil	[48] [54] [46] [52] [53] [37,40] [39]
eudesm-5,7-dien (**3**)	*G. guidonia*	Leaf essential oil Leaves	[49,50] [60]
Eudesm-4,11-diene (**4**)	*G. guidonia*	Leaf essential oil Leaves	[49,50] [60]
5α,6α-epoxy-eudesm-7-ene (**5**)	*G. guidonia*	Leaf essential oil	[50]
Eudesm-6-en-4β-ol (**6**)	*G. guidonia*	Leaf essential oil Leaves	[49,50] [60]
Guai-6-en-10β-ol (**7**)	*G. guidonia* *G. macrophylla*	Leaf essential oil Leaves Leaf essential oil Stem bark essential oil Wood	[49,50] [52,62] [40,92] [38] [53]
Eudesm-5,7-dien-2α-ol (**8**)	*G. guidonia*	Leaf essential oil	[49,50]
5α,6α-epoxy-eudesm-7-en-9-ol (**9**)	*G. guidonia*	Leaf essential oil	[50]
5α,6α,7α,8α-diepoxy-eudesmane (**10**)	*G. guidonia*	Leaf essential oil Leaves	[50] [60]
Viridiflorol (**11**) 3-oxo-10-alloaromadendranol (**12**) Voleneol (**13**) Alismol (**14**) Alismoxide (**15**) (-)-4β,10α-aromadendranediol (**16**) Palustrol (**17**) Ledol (**18**)	*G. guidonia* *G. macrophylla* *G. guidonia* *G. guidonia* *G. kunthiana* *G. kunthiana* *G. kunthiana* *G. macrophylla* *G. macrophylla* *G. macrophylla*	Wood bark Branch essential oil Stem bark essential oil Stem bark essential oil Wood bark Wood bark Leaves Leaves Leaves Wood Leaves Leaf essential oil Leaves Leaf essential oil Stem bark essential oil	[47] [44] [43] [38] [47] [47] [54] [54] [54] [53] [52] [37,40,92] [52] [37,40,92] [38]
(2S*)-eudesm-5,7-dien-2-ol (**19**) Caryophyllene oxide (**20**) 6α-ethoxyeudesm-4(15)-en-1β-ol (**21**) (7R*)-5-*epi*-opposit-4(15)-ene-1β,7-diol (**22**) Eudesm-4(15)-ene-1β,6α-diol (**23**) 5-*epi*-eudesm-4(15)-ene-1β,6β-diol (**24**) Eudesm- 4(15)-ene-1β,5α-diol (**25**) Eudesm-4(15),7-dien-1β-ol (**26**) (7R*)-opposit-4(15)-ene-1β,7-diol (**27**) α-cubebene (**28**) α-ylangene (**29**) α-copaene (**30**) α-gurjunene (**31**) α-humulene (**32**) β-caryophyllene (**33**) Allo-aromadendrene (**34**) Germacrene D (**35**)	*G. guidonia* *G. macrophylla* *G. cedrata* *G. guidonia* *G. guidonia* *G. guidonia* *G. guidonia* *G. guidonia* *G. guidonia* *G. guidonia* *G. guidonia* *G. macrophylla* *G. guidonia* *G. macrophylla* *G. cedrata* *G. macrophylla* *G. guidonia* *G. cedrata* *G. kunthiana* *G. macrophylla* *G. macrophylla* *G. guidonia* *G. macrophylla* *G. guidonia* *G. cedrata* *G. macrophylla* *G. guidonia* *G. cedrata* *G. macrophylla* *G. guidonia* *G. kunthiana*	Leaves Wood Bark essential oil Branch essential oil Stem bark essential oil Seeds Seeds Seeds Seeds Seeds Seeds Seeds Leaf essential oil Stem bark essential oil Fruit essential oil Leaf essential oil Leaf essential oil Bark essential oil Leaf essential oil Stem bark essential oil Fruit essential oil Leaf essential oil Bark essential oil Leaf essential oil Leaf essential oil Leaf essential oil Stem bark essential oil Fruit essential oil Branch essential oil Stem bark essential oil Leaf essential oil Stem bark essential oil Fruit essential oil Leaf essential oil Branch essential oil Stem bark essential oil Bark essential oil Leaf essential oil Stem bark essential oil Fruit essential oil Leaf essential oil Bark essential oil Leaf essential oil Fruit essential oil Branch essential oil Stem bark essential oil	[60] [36] [44] [43] [53] [51] [51] [51] [51] [51] [51] [51] [37,40,92] [38] [39] [49] [37,40,41,92] [36] [37,40,92] [38] [39] [49] [36] [46] [37,40,92] [37,40,41,92] [38] [39] [44] [43] [37,40,41,92] [38] [39] [49] [44] [43] [36] [37,40,92] [38] [39] [49] [36] [37,40,92] [39] [44] [43]
Bicyclogermacrene (**36**) γ-cadinene (**37**) δ-cadinene (**38**) Germacrene-D-4-ol (**39**) Aromadendrene (**40**) *cis*-bicyclogermacradiene (**41**) Viridiflorene (**42**) *trans*-bicyclogermacradiene (**43**) *cis*-calamenene (**44**) Globulol (**45**) *cis*-cubenol (**46**) *trans*-cubenol (**47**) δ-elemene (**48**) β -elemene (**49**) β-cubebene (**50**) α-guaiene (**51**) γ-muurolene (**52**) 5,6,7,8-diepoxy-eudesmane (**53**) Cadina-1(6),4-diene (**54**) *cis*-β-guaiene (**55**) β-bisabolene (**56**) β-cadinene (**57**) Cadina-1,4-diene (**58**) *epi*-globulol (**59**) 1-*epi*-cubenol (**60**) τ-cadinol (**61**) τ-muurolol (**62**) α-cadinol (**63**) α-cadinene (**64**) 1-cubenol (**65**) Longifolene (**66**) α-muurolene (**67**) γ-elemene (**68**) α-eudesmol (**69**) Isocaryophyllene oxide (**70**) α-muurolol (**71**) 6,7-epoxy-2,9-humuladiene (**72**) E-caryophyllene (**73**) α-bergamotene (**74**) β-farnesene (**75**) α-curcumene (**76**) α-zingiberene (**77**) Calamenene (**78**) β-sesquiphellandrene (**79**) Cadalene (**80**) β-copaene (**81**) 9-epi-β-caryophyllene (**82**) γ-amorphene (**83**) Germacrene A (**84**) γ-eudesmol (**85**) 1(10)-epoxy-4,7-humuladiene (**86**) 1(10),4-diepoxy-7-humulene (**87**) alloaromadendrane-4α,10β-diol (**88**) *trans*-4,10(14)-cadinadiene (**89**) cyclosativene (**90**) 6,9-guaiadiene (**91**) γ-himachalene (**92**) α-amorphene (**93**) *trans*-muurola-4(14),5-diene (**94**) *trans*-β-guaiene (**95**) δ-amorphene (**96**) α-calacorene (**97**) Selina-3,7(11)-diene (**98**) Elemol (**99**) Germacrene B (**100**) β-calacorene (**101**) Guaiol (**102**) 1,10-di-epi-cubenol (**103**) Isolongifolan-7-α-ol (**104**) α-acorenol (**105**) *cis*-cadin-4-en-7-ol (**106**) Hinesol (**107**) Cedr-8(15)-en-9α-ol (**108**) Valerianol (**109**) 7-epi-α-eudesmol (**110**) β-bourbonene (**111**) *cis*-caryophyllene (**112**) α-cis-bergamoteme (**113**) α-santalene (**114**) β-gurjunene (**115**) β-santalene (**116**) Drima-7,9(11)-diene (**117**) α-selinene (**118**) (E)-iso-γ-bisabolene (**119**) Caryophyllene epoxide (**120**) *trans*-nerolidol (**121**) Humulene epoxide II (**122**) *epi*-α-cadinol (**123**) β-eudesmol (**124**) Mustakone (**125**) α-*trans*-bergamotene (**126**)	*G. kunthiana* *G. macrophylla* *G. macrophylla* *G. guidonia* *G. macrophylla* *G. guidonia* *G. macrophylla* *G. macrophylla* *G. macrophylla* *G. macrophylla* *G. cedrata* *G. macrophylla* *G. macrophylla* *G. macrophylla* *G. cedrata* *G. macrophylla* *G. macrophylla* *G. guidonia* *G. guidonia* *G. macrophylla* *G. guidonia* *G. macrophylla* *G. guidonia* *G. guidonia* *G. macrophylla* *G. cedrata* *G. guidonia* *G. macrophylla* *G. macrophylla* *G. macrophylla* *G. macrophylla* *G. macrophylla* *G. cedrata* *G. macrophylla* *G. cedrata* *G. macrophylla* *G. macrophylla* *G. cedrata* *G. macrophylla* *G. macrophylla* *G. macrophylla* *G. macrophylla* *G. cedrata* *G. cedrata* *G. cedrata* *G. macrophylla* *G. cedrata* *G. cedrata* *G. guidonia* *G. guidonia* *G. guidonia* *G. kunthiana* *G. kunthiana* *G. kunthiana* *G. kunthiana* *G. kunthiana* *G. kunthiana* *G. kunthiana* *G. kunthiana* *G. macrophylla* *G. macrophylla* *G. macrophylla* *G. macrophylla* *G. macrophylla* *G. guidonia* *G. guidonia* *G. macrophylla* *G. guidonia* *G. macrophylla* *G. macrophylla* *G. macrophylla* *G. macrophylla* *G. macrophylla* *G. macrophylla* *G. macrophylla* *G. macrophylla* *G. macrophylla* *G. macrophylla* *G. macrophylla* *G. macrophylla* *G. macrophylla* *G. macrophylla* *G. macrophylla* *G. macrophylla* *G. macrophylla* *G. macrophylla* *G. macrophylla* *G. macrophylla* *G. macrophylla* *G. scabra* *G. scabra* *G. scabra* *G. convergens* *G. scabra* *G. convergens* *G. convergens* *G. convergens* *G. silvatica* *G. humatensis* *G. scabra* *G. silvatica* *G. scabra* *G. silvatica* *G. silvatica* *G. scabra*	Stem bark essential oil Leaf essential oil Leaf essential oil Leaf essential oil Fruit essential oil Leaf essential oil Branch essential oil Leaf essential oil Stem bark essential oil Leaf essential oil Branch essential oil Stem bark essential oil Leaf essential oil Fruit essential oil Stem bark essential oil Stem bark essential oil Stem bark essential oil Fruit essential oil Bark essential oil Stem bark essential oil Stem bark essential oil Fruit essential oil Stem bark essential oil Fruit essential oil Bark essential oil Stem bark essential oil Stem bark essential oil Leaf essential oil Leaf essential oil Branch essential oil Stem bark essential oil Leaf essential oil Leaf essential oil Fruit essential oil Leaf essential oil Leaf essential oil Branch essential oil Stem bark essential oil Fruit essential oil Leaf essential oil Bark essential oil Leaf essential oil Fruit essential oil Fruit essential oil Fruit essential oil Fruit essential oil Fruit essential oil Bark essential oil Fruit essential oil Bark essential oil Fruit essential oil Leaf essential oil Fruit essential oil Leaf essential oil Bark essential oil Fruit essential oil Leaf essential oil Fruit essential oil Leaf essential oil Leaf essential oil Bark essential oil Bark essential oil Bark essential oil Leaf essential oil Bark essential oil Bark essential oil Branch essential oil Stem bark essential oil Branch essential oil Stem bark essential oil Leaf essential oil Leaf essential oil Leaf essential oil Leaf essential oil Leaf essential oil Leaf essential oil Leaf essential oil Leaf essential oil Leaf essential oil Leaf essential oil Leaf essential oil Leaf essential oil Leaf essential oil Wood bark Wood bark Wood Stem bark essential oil Leaf essential oil Leaf essential oil Leaf essential oil Leaf essential oil Leaf essential oil Leaf essential oil Leaf essential oil Leaf essential oil Leaf essential oil Leaf essential oil Leaf essential oil Leaf essential oil Leaf essential oil Leaf essential oil Leaf essential oil Leaf essential oil Leaf essential oil Leaf essential oil Leaf essential oil Leaf essential oil Leaf essential oil Leaf essential oil Leaf essential oil Leaf essential oil Branch essential oil Leaf essential oil Branch essential oil Branch essential oil Branch essential oil Branch essential oil Branch essential oil Leaf essential oil Branch essential oil Leaf essential oil Branch essential oil Branch essential oil Leaf essential oil	[43] [46] [37,40,92] [37,40,41,92] [39] [49] [44] [37,41] [38] [49] [44] [43] [37,40,92] [39] [38] [38] [38] [39] [36] [38] [38] [39] [38] [39] [36] [38] [38] [49] [49] [44] [43] [41] [49] [39] [49] [49] [44] [43] [39] [41] [36] [49] [39] [39] [39] [39] [39] [36] [39] [36] [39] [40] [39] [40] [36] [39] [40] [39] [40] [40] [36] [36] [36] [41] [36] [36] [44] [43] [44] [43] [46] [46] [46] [46] [46] [46] [46] [46] [41] [41] [41] [41] [41] [47] [47] [53] [43] [42] [42] [42] [42] [42] [42] [42] [42] [42] [42] [42] [42] [42] [42] [42] [42] [42] [42] [42] [42] [42] [45] [45] [45] [45] [45] [45] [45] [45] [45] [45] [45] [45] [45] [45] [45] [45]
Diterpenoid			
Cneorubin A (**127**) Cneorubin B (**128)** Cneorubin X (**129**) Cneorubin Y (**130**) Isopimara-7,15-dien-2α,3β-diol (**131**) Isopimara-7,15-dien-3β-ol (**132**) 3-oxo-labd-8(17),12*Z*,14-triene (**133**) 3α-hydroxylabd-8(17),12*Z*,14-triene (**134**) 3β-hydroxylabd-8(17),12*Z*,14-triene (**135**) (-)-2-oxo-13-hydroxy,3,14-clerodandiene (**136**) 19-hydroxymanoyloxide (**137**) 13-hydroxy-3,14-clerodandiene (**138**) *ent*-kaur-16-en-2-one (**139**) *ent*-kaur-16-ene (**140**) *ent*-3β-hydroxykaur-16-ene (**141**) *ent*-3α-hydroxykaur-16-ene (**142**) Kolavelool (**143**) Kolavenol (**144**) Kolavenal (**145**) (-)-nephthenol (**146**) *ent*-13-epimanoyloxide (**147**) 7α-hydroperoxy-isopimara-8(14),15-diene-2α,3β-diol (**148**) 19-nor-isopimara-7,15,4(18)-trien-3-one (**149**) Isopimara-7,15-dien-3-one (**150**) Isopimara-7,15-dien-2β-ol (**151**) Isopimara-7,15-dien-2α-ol (**152**) Manoyl oxide (**153**) Labda-8,14-dien-13-ol (**154**) phytol (**155**) *ent*-8(14),15-sandaracopimaradiene-2α,18-diol (**156**) *ent*-8(14),15-sandaracopimaradine-2β,18-diol (**157**) Isopimara-7,15-diene (**158**) Labda-8,13-(*E*)-dien-15-ol (**159**) Boscartol C (**160**) 13-*epi*-dolabradiene (**161**) Phyllocladane (**162**) Sandaracopimarinal (**163**) Kaurene (**164**)	*G. guidonia* *G. guidonia* *G. guidonia* *G. guidonia* *G. macrophylla* *G. macrophylla* *G. trichilioides* *G. trichilioides* *G. trichilioides* *G. trichilioides* *G. trichilioides* *G. trichilioides* *G. kunthiana* *G. kunthiana* *G. kunthiana* *G. kunthiana* *G. kunthiana* *G. kunthiana* *G. kunthiana* *G. kunthiana* *G. kunthiana* *G. macrophylla* *G. macrophylla* *G. macrophylla* *G. macrophylla* *G. macrophylla* *G. macrophylla* *G. macrophylla* *G. macrophylla* *G. guidonia* *G. rhophalocarpa* *G. rhophalocarpa* *G. macrophylla* *G. macrophylla* *G. guidonia* *G. macrophylla* *G. macrophylla* *G. macrophylla* *G. silvatica*	Leaves The aerial parts Leaves The aerial parts Leaves The aerial parts Leaves Leaves Leaves Leaf essential oil Leaves Leaves Leaves Leaves Leaves Leaves Leaves Leaves Leaves Leaves Leaves Leaves Leaves Leaves Leaves Leaves Leaves Leaves Leaf essential oil Leaves Leaves Leaf essential oil Leaves Leaf essential oil Stem bark essential oil Leaves Leaves Leaves Leaves Leaves Leaf essential oil Leaves Leaf essential oil The aerial parts Leaf essential oil Leaf essential oil Leaf essential oil Leaf essential oil	[48] [58] [48] [58] [48] [58] [48] [56] [55,56] [37,40,92] [57] [57] [57] [57] [57] [57] [54] [54] [54] [54] [54] [54] [54] [54] [54] [55] [55] [52,55] [37,40,92] [52] [55] [40,92] [52,55] [37,40,92] [38] [55] [55] [60] [59] [59] [37,40,92] [52] [37,40,92] [58] [42] [42] [42] [45]
Triterpenoid			
3,4-secotirucalla-4(28),7,24-trien-3,21-dioic-acid (**165**) 3,4-secotirucalla-4(28),7,24-trien-3,21-dioic-acid-3-methyl ester (**166**) 3β-*O*-tigloylmelianol (**167**) 23-hydroxy-5α-lanosta7,9(11),24-triene-3-one (**168**) 5α-lanosta-7,9(11),24-triene-3α,23-diol (**169**) cycloart-23*E*-ene-3β,25-diol (**170**) (23S*,24S*)-dihydroxycicloart-25-en-3-one (**171**) Glabretal (**172**) Cycloart-24-en-3,23-dione (**173**) 23-hydroxycycloart-24-en-3-one(epimers) (**174** & **175**) 3β-hydroxycycloart-24-en-23-one (**176**) 25-hydroxycycloart-23-en-3-one (**177**) 3β-21-dihydroxycycloartane (**178**) 3β,21,22,23tetrahydroxycycloartane-24(31),25-diene (**179**) 21,24-epoxy-3α,7α,21,23tetraacetoxy-25-hydroxy-4α,4β,8β-trimethyl-14,18-cyclo-5α,13α,14α,17α-cholestane (**180**) 21,23-epoxy-3α,7α,21,24,25pentaacetoxy-4α,4β,8β-trimethyl-14,18-cyclo-5α,13α,14α,17α-cholestane (**181**) 24-acetoxy-25-hydroxy-3,7-dioxoapotirucalla-14-en-21,23-olide (**182**) 7α,24,25-trihydroxy-3-oxoapotirucalla-14-en-21,23-olide (**183**) Melianone (**184**) Melianodiol (**185**) Guareolide (**186**) Guareoic acid A (**187**) Guareoic acid B (**188**) Flindissone (**189**) Picroquassin E (**190**) 21-α-acetylmelianone (**191**) cycloarta-23,25-dien-3-one (**192**) (23S*)-cycloart-24-ene-3β,23-diol (**193**) (23R*)-cycloart-24-ene-3β,23-diol (**194**) Meliantriol (**195**) 22,25-dihydroxycycloart-23E-en-3-One (**196**) 24-methylenecycloartane-3β,22-diol (**197**) 3β-*O*-tigloylmeliantriol (**198**) Melianol (**199**)	*G. cedrata* *G. cedrata* *G. kunthiana* *G. rhophalocarpa* *G. rhophalocarpa* *G. macrophylla* *G. humaitensis* *G. macrophylla* *G. glabra* *G. trichilioides* *G. macrophylla* *G. guidonia* *G. trichilioides* *G. macrophylla* *G. trichilioides* *G. macrophylla* *G. guidonia* *G. trichilioides* *G. macrophylla* *G. trichilioides* *G. trichilioides* *G. jamicensis* *G. jamicensis* *G. convergens* *G. convergens* *G. convergens* *G. grandiflora* *G. convergens* *G. grandiflora* *G. kunthiana* *G. guidonia* *G. guidonia* *G. guidonia* *G. guidonia* *G. guidonia* *G. grandiflora* *G. macrophylla* *G. guidonia* *G. guidonia* *G. kunthiana* *G. macrophylla* *G. macrophylla* *G. kunthiana* *G. kunthiana*	Bark Bark Fruits Leaves Leaves Leaves wood Leaves Heartwood Leaves Leaves Leaves Leaves Leaves Leaves Leaves Leaves Leaves Leaves Leaves Leaves Leaves and twigs Leaves and twigs Leaves and branches Leaves and branches Leaves and branches Seeds Leaves and branches Seeds The aerial parts The aerial parts The aerial parts The aerial parts The aerial parts The aerial parts Seeds Leaves Leaves wood Leaves Wood The aerial parts Leaves Leaves Fruits Fruits	[33] [33] [91] [59] [59] [56,62] [53] [56] [63] [61] [62] [60] [61] [62] [61] [62] [60] [61] [62] [61] [61] [64] [64] [67] [67] [67] [65] [67] [65] [87] [58] [58] [58] [58] [58] [65] [52,62] [60] [53] [60] [53] [87] [62] [62] [66] [66]
Limonoid			
7-deacetoxy-7-oxogedunin (**200**) Gedunin (**201**) Chisomicine D (**202**) Chisomicine E (**203**) Chisomicine F (**204**) 3-(2′hydroxyisovaleroyl) khasenegasin I (**205**) Methyl-6-acetoxyangolensate (**206**) Dregeanin (**207**) Mombasol (**208**) 6α-acetoxygedunin (**209**) 14,15β-epoxyprieuriani (**210**) Humilinolide E (**211**) Methyl-2-hydroxy-3β-tigloyloxy-1-oxomeliac-8(30)-enate (**212**) Swietenine acetate (**213**) Methyl angolensate (**214**) 2’-hydroxyrohitukin (**215**) 7-acetyldihydronomilin (**216**) Ecuadorin (**217**) 7-oxo-gedunin (**218**) Prieurianin (**219**) Fissinolide (**220**) Dihydrogedunin (**221**) Mayombensin (**222**) Azadirachtin I (**223**) Angustinolide (**224**)	*G. grandiflora* *G. grandiflora* *G. guidonia* *G. guidonia* *G. guidonia* *G. guidonia* *G. thompsonii* *G. thompsonii* *G. guidonia* *G. grandiflora* *G. guidonia* *G. kunthiana* *G. kunthiana* *G. kunthiana* *G. kunthiana* *G. cedrata* *G. guidonia* *G. kunthiana* *G. guidonia* *G. guidonia* *G. guidonia* *G. thompsonii* *G. mayombensis* *G. mayombensis* *G. trichilioides*	Seeds Seeds Stem bark Stem bark Stem bark Stem bark Bark Bark Bark Seeds Root Bark Fruits Fruits Fruits Fruits Bark The aerial parts Aerial parts Root bark Root bark Seeds Heartwood Twigs Twigs Seeds	[65] [65] [76] [76] [76] [76] [70] [70] [47] [65] [73] [71] [71] [71] [71] [33] [58] [72] [73] [73] [74] [34] [77] [77] [75]
Other Compounds			
Quercetin 3-*O*-β-D-glucopyranoside (**225**) Quercetin 3-*O*-β-D-galactopyranoside (**226**) Kaempferol-7-*O*-β-D-glucopyranoside (**227**) Dehydrodiconiferyl-alcohol-4-β-d-glucoside (**228**) β-sitosterol (**229**) Stigmasterol (**230**) Stigmasterol glucoside (**231**) β-sitosterol glucoside (**232**) β-sitostenone (**233**) 2α,3β-dihydroxy-16,17-seco-pregn-17-ene-16-oic acid methyl ester 2β,19-hemiketal (**234**) 2,3:16,17-di-seco-pregn-17-ene-3-oic-acid-16-oic acid methyl ester-19-hydroxy-2-carboxylic acid-2,19-lactone (**235**) Ergosta-5,24(24′)-diene-3β,7α,21-triol (**236**) Ergosta-5,24(24′)-diene-3β,4β,22S-triol (**237**) Ceramide A (**238**) Ceramide B (**239**) Scopoletin (**240**)	*G. macrophylla* *G. macrophylla* *G. macrophylla* *G. macrophylla* *G. glabra* *G. cedrata* *G. convergens* *G. trichilioides* *G. guidonia* *G. convergens* *G. mayombensis* *G. mayombensis* *G. glabra* *G. guidonia* *G. guidonia* *G. convergens* *G. convergens* *G. mayombensis* *G. mayombensis* *G. rhopalocarpa*	Flowering branches Flowering branches Flowering branches Flowering branches Heartwood Heartwood Leaves and branches Seeds and bark Leaves Leaves and branches Twigs Twigs Heartwood Trunk bark Trunk bark Leaves and branches Leaves and branches Twigs Twigs Leaves	[80] [80] [80] [80] [78] [93] [67] [75] [48,60] [67] [77] [77] [78] [79] [79] [67] [67] [77] [77] [59]

**Table 2:** Bioactivities of *Guarea* Genus.

Biology Activity	Compound or Extract	Plant Species	Ref.
**Cytotoxic**: Compounds **210** and **219** are active against leukemia cell line P388 ED_50_ 0.47–0.74 µg/mL and P388 ED50 4.4–7.8 µg/mL; methylene chloride extract evaluated against U-937 cell lines with each LD_50_ of 6.1 ± 0.5 µg/mL and 6.1 ± 1.2 µg/mL while the seed of *G. guidonia* had LD_50_ of 28.8 ± 8.2 µg/mL; **156**, **157**, **168**, **169**, **230**, and **240** were tested against the KB cell line with IC_50_ of 48; 75.8; 30.2, 21.2; > 1272; and 130.2 µM, respectively; **170** was tested with EC_50_ HL-60 (18.3), HeLa (52.1), B16F10-Nex2 (58.9), A2058 (60.7), and MCF-7 (63.5) µM while **131** and **132** against five cell lines over 100 µM; **189** showed activity with EC_50_ 25, 27, 50, and > 100 µM for the Jurkat, HeLa, MCF-7, and PBMC cell lines; **187** with EC_50_ 39 µM against the Jurkat cell line; **202** (U-937 IC_50_ 20 ± 3 µM and HeLa > 50 µM. **Anti-inflammation:** Anti-inflammation against male Wistar rats showed the effects of 8.0 mL/kg extract dose and the effects increased from time to time by 5.0 mL/kg extract. **Antimalarial**: Three extracts have IC_50_ 50 µg/mL from petroleum ether extract of leaves, methanol extract of stem bark and fruits, and also chloroform extract of stem bark. **Anti-parasitic** **Antiprotozoal**: Methylene chloride extract of bark and leaves *G. polymera* has a selectivity index against *Leishmania Viannia panamensis* LD_50_/ED_50_ 1.5 µg/mL and the seeds of *G. guidonia* have activity against *Plasmodium falciparum* with LD_50_/IC_50_ 2.9 µg/mL (IC_50_ **156** (16.8); **157** (49.7); **168** (7.2) µg/mL. **Antiviral** **Antimicrobial**: The essential oil has been evaluated for MIC and MBC against *S. infantris, S. tyrphimurium* and *S. give* with MIC and MBC 54.6 µg/mL. **Insectisidal activity**: The ethyl acetate extract against *Aedes aegyptyi* had LC_50_ and LC_90_ 105.7 and 408.9 µg/mL; **185** with LC_50_ 14.4 and LC_90_ 17.54; and **195** over 100 µg/mL. **Antioxidant**: The essential oil, alcoholic, aqueous and ethyl acetate extracts showed IC_50_ 15.3; 176.8 µg/mL **Phosphorylation inhibitor**	Cycloart-23*E*-ene-3β,25-diol (**170**) (23S*,24S*)-dihydroxycicloart-25-en-3-one (**171**) Isopimara-7,15-dien-2α,3β-diol (**131**) Isopimara-7,15-dien-3β-ol (**132**) Guareolide (**186**) Guareoic acid A (**187**) Guareoic acid B (**188**) Flindissone (**189**) Picroquassin E (**190**) 14,15β-epoxyprieuriani (**210**) 7-oxo-gedunin (**218**) Prieurianin (**219**) Chisomicine D (**202**) Chisomicine E (**203**) Chisomicine F (**204**) 3-(2′-hydroxyisovaleroyl)khasenegasin I (**205**) *ent*-8(14),15-sandaracopimaradiene-2α,18-diol (**156**) *ent*-8(14),15-sandaracopimaradine-2β,18-diol (**157**) 23-hydroxy-5α-lanosta 7,9(11),24-triene-3-one (**168**) 5α-lanosta-7,9(11),24-triene-3α,23-diol (**169**) Stigmasterol (**230**) Scopoletin (**240**) Methylene chloride extract Methylene chloride extract Ethanol extract Petroleum Extract Methanol Extract Water Extract Chloroform Extract Hexane extract *ent*-8(14),15-sandaracopimaradiene-2α,18-diol (**156**) *ent*-8(14),15-sandaracopimaradine-2β,18-diol (**157**) 23-hydroxy-5α-lanosta 7,9(11),24-triene-3-one (**168**) 5α-lanosta-7,9(11),24-triene-3α,23-diol (**169**) Stigmasterol (**230**) Scopoletin (**240**) Methylene chloride extract Methylene chloride extract Methanol extract 3β-*O*-tigloylmelianol (**167**) Aqueous Extract Essential oil Methanol Extract Melianone (**184**) Melianodiol (**185**) 21-α-acetylmelianone (**191**) 6α-acetoxygedunin (**209**) Aqueous extract Acetate extract Alcoholic extract Essential oil Ethyl acetate phase Melianodiol (**185**) Meliantriol (**195**) Essential oil Alcoholic extract Aqueous extract Ethyl acetate extract 7-deacetoxy-7-oxogedunin (**200**) Gedunin (**201**)	*G. macrophylla* *G. macrophylla* *G. macrophylla* *G. macrophylla* *G. guidonia* *G. guidonia* *G. guidonia* *G. guidonia* *G. guidonia* *G. guidonia* *G. guidonia* *G. guidonia* *G. guidonia* *G. guidonia* *G. guidonia* *G. guidonia* *G. rhophalocarpa* *G. rhophalocarpa* *G. rhophalocarpa* *G. rhophalocarpa* *G. rhophalocarpa* *G. rhophalocarpa* *G. guidonia* *G. polymera* L *G. guidonia* *G. multiflora* *G. multiflora* *G. multiflora* *G. multiflora* *G. kunthiana* *G. rhophalocarpa* *G. rhophalocarpa* *G. rhophalocarpa* *G. rhophalocarpa* *G. rhophalocarpa* *G. rhophalocarpa* *G. guidonia* *G. polymera* L *G. polymera* L *G. kunthiana* *G. guidonia* *G. kunthiana* *G. kunthiana* *G. grandiflora* *G. grandiflora* *G. grandiflora* *G. grandiflora* *G. kunthiana* *G. kunthiana* *G. kunthiana* *G. kunthiana* *G. kunthiana* *G. kunthiana* *G. kunthiana* *G. kunthiana* *G. kunthiana* *G. kunthiana* *G. kunthiana* *G. grandiflora* *G. grandiflora*	[56] [56] [56] [56] [58] [58] [58] [58] [58] [73] [73] [73] [76] [76] [76] [76] [59] [59] [59] [59] [59] [59] [90] [90] [82] [83] [83] [83] [83] [84] [59] [59] [59] [59] [59] [59] [90] [90] [90] [91] [85] [88] [88] [65] [65] [65] [65] [88] [88] [88] [88] [88] [87] [87] [87] [88] [88] [88] [89] [89]

## Data Availability

The study did not report any data.
